# Metabolic imaging and secondary ion mass spectrometry to define the structure and function of liver with acute and chronic pathology

**DOI:** 10.1117/1.JBO.25.1.014508

**Published:** 2019-12-17

**Authors:** Daria Kuznetsova, Svetlana Rodimova, Alexander Gulin, Dmitry Reunov, Nikolai Bobrov, Anastasia Polozova, Alexander Vasin, Vladislav Shcheslavskiy, Natalia Vdovina, Vladimir Zagainov, Elena Zagaynova

**Affiliations:** a Privolzhsky Research Medical University, Institute of Experimental Oncology and Biomedical Technologies, Nizhny Novgorod, Russia; b Russian Academy of Sciences, N.N. Semenov Federal Research Center for Chemical Physics, Moscow, Russia; c Lomonosov Moscow State University, Department of Chemistry, Moscow, Russia; d Federal Medical and Biological Agency, Volga District Medical Centre, Nizhny Novgorod, Russia; e Becker & Hickl GmbH, Berlin, Germany

**Keywords:** metabolic imaging, fluorescence lifetime imaging, multiphoton microscopy, time-of-flight secondary ion mass spectrometry, liver

## Abstract

Conventional techniques are insufficient precisely to describe the internal structure, the heterogeneous cell populations, and the dynamics of biological processes occurring in diseased liver during surgery. There is a need for a rapid and safe method for the successful diagnosis of liver disease in order to plan surgery and to help avoid postoperative liver failure. We analyze the progression of both acute (cholestasis) and chronic (fibrosis) liver pathology using multiphoton microscopy with fluorescence lifetime imaging and second-harmonic generation modes combined with time-of-flight secondary ion mass spectrometry chemical analysis to obtain new data about pathological changes to hepatocytes at the cellular and molecular levels. All of these techniques allow the study of cellular metabolism, lipid composition, and collagen structure without staining the biological materials or the incorporation of fluorescent or other markers, enabling the use of these methods in a clinical situation. The combination of multiphoton microscopy and mass spectrometry provides more complete information about the liver structure and function than could be assessed using either method individually. The data can be used both to obtain new criteria for the identification of hepatic pathology and to develop a rapid technique for liver quality analysis in order to plan surgery and to help avoid postoperative liver failure in clinic.

## 1 Introduction

The great challenge in liver surgery is of pathological changes to the liver that may lead to a reduction of regenerative potential in diseased liver tissue. The main methods to assess both liver quality and the pathological changes include ultrasound, positron emission tomography (PET), magnetic resonance imaging (MRI), computed tomography (CT), biochemical blood analysis, morphological and immunohistochemical analysis of biopsy specimens, and clearance tests using indocyanine, as well as various radioactive isotopes.^1^^–^^3^ Ultrasound, PET, MRI, and CT are quite informative, but they only provide information about the liver tissue at the level of the whole organ, not allowing resolution at a cellular level. Biochemical blood analysis is among the simplest methods for studying liver function.^4^ Most of the indices are not organ-specific and may not reflect the true functional state of the liver. Moreover, these figures in most cases do not change in focal liver diseases since there are no pathological disorders in the healthy liver parenchyma. The morphological and immunohistochemical analysis of biopsy specimens requires additional staining with both standard histological dyes and specific, expensive antibodies to detect particular markers.^5^ There is also the likelihood of nonspecific background staining, which may give false results. Clearance tests are based on an assessment of the rate of removal of various substances from biological fluids or body tissues, and can characterize the absorption and excretory function of the liver.^1^ However, they do not provide information about the processes occurring in the liver tissues at the level of individual hepatocytes. Thus, these conventional techniques are insufficient to describe precisely the internal structure, heterogeneous cell populations, and the dynamics of the biological processes of the diseased liver during surgery. There is a need for a rapid and safe method for the successful diagnosis of liver disease in order to plan surgery and to avoid postoperative liver failure.

Currently, optical bioimaging and microstructural analysis are being actively implemented in various areas of biomedical research.^6^ Multiphoton microscopy with fluorescence lifetime imaging (FLIM) and second-harmonic generation (SHG) modes together with composition analysis by time-of-flight secondary ion mass spectrometry (TOF-SIMS) are of particular interest to the study of liver tissue. While multiphoton microscopy enables quantitative deep imaging of the physiology, morphology, and cell–cell interactions of the intact tissue of live animals with high resolution, FLIM allows interrogation of local microenvironment of the fluorophores and evaluate its pH, refractive index, temperature, and viscosity since fluorescence lifetime depends on them.^7^^,^^8^ Thus FLIM can add molecular specificity to the structural information. Moreover, it enables identification of different endogenous and exogenous fluorophores that have overlapping or poorly defined spectra but that have different fluorescence lifetimes.^9^^,^^10^

TOF-SIMS is a sensitive, label-free technique used for both routine composition analysis and for chemical mapping of cells and tissues.^11^^–^^14^ By comparing the spectra of control and affected tissues, differences in the chemical composition can be revealed.^15^^,^^16^ Combined with Fourier transform infrared spectroscopy, TOF-SIMS has been successfully used for fatty liver investigations of patients’ liver resection specimens. The accumulation of triacylglycerols, diacylglycerols, monoacylglycerols, and fatty acids, with the apparition of myristic acid, together with a dramatic depletion of vitamin E has been observed compared with normal liver samples.^17^^,^^18^ Applying TOF-SIMS, Murayama et al.^19^ showed that acetaminophen-overdosed mouse liver displayed chemical changes mainly corresponding to glycogen depletion on a subcellular scale. Multiphoton FLIM microscopy and mass spectrometry do not need additional staining of samples or the incorporation of any markers to study metabolism (based on the fluorescence of the dehydrogenase cofactors NADH, NADPH, and FAD), lipid composition (based on the secondary ion spectrum), and fibrous structures (based on second optical harmonic generation by collagen). These parameters display specific changes in liver hepatocytes suffering common pathological diseases.^20^^,^^21^ As a continuation of our previous works,^22^^,^^23^ here we analyze acute and chronic liver pathology during the progression of pathological changes in hepatocytes at the cellular and molecular level, using multiphoton microscopy and mass spectrometry to obtain new data. The data can be used to develop new criteria for the identification of hepatic pathology, aimed at providing rapid analysis of liver quality in order to plan surgery and to avoid postoperative liver failure.

## 2 Materials and Methods

### 2.1 Animal Models

All *in vivo* experiments and experimental protocols were approved by the research ethics board of the Privolzhsky Research Medical University, Nizhny Novgorod, Russia. The experiments were performed on male Wistar rats with a body weight of 300 to 400 g. We modeled both acute and chronic liver pathology: cholestasis and fibrosis.

Acute cholestasis was induced by bile duct ligation. This experimental model is well accepted and used worldwide in hundreds of laboratories to induce liver cholestasis. It results in intrahepatic biliary epithelial cell proliferation and myofibroblastic differentiation of the portal fibroblasts around the proliferating biliary epithelial cells.^24^ Bile duct ligation was performed after midline laparotomy. The common bile duct was ligated two times with 5–0 silk. The surgical procedures were performed under aseptic conditions. Body temperature was controlled by placing the animals on a heating pad set to 37°C. Imaging was performed 1 and 3 weeks after bile duct ligation. Healthy rat livers served as controls. Each group consisted of 5 rats.

Liver fibrosis (models using chronic-plus-multiple binges of ethanol) was induced by intragastric infusion of a solution containing ethanol as described in Ref. 25. Imaging was performed 4, 8, and 12 weeks after fibrosis induction. Healthy rat livers served as controls. Each group consisted of 5 rats.

### 2.2 Multiphoton Fluorescence Microscopy with FLIM and SHG

The two-photon excited fluorescence intensity (TPEF), the SHG of collagen fibers, and FLIM images of NAD(P)H and FAD were obtained using an LSM 880 (Carl Zeiss, Germany) laser scanning confocal microscope equipped with a time-correlated single-photon counting system (Simple-Tau 152, Becker & Hickl GmbH, Germany). NAD(P)H and FAD fluorescence were excited with a Ti:Sa femtosecond laser, using an 80-MHz repetition rate and a pulse duration of 140 fs at the wavelengths of 750 and 900 nm, respectively. Emission was detected in the ranges of 450 to 500 nm for NAD(P)H and 500 to 550 nm for FAD. An average of 10,000 photons were collected per decay curve. The average power of the Ti:Sa laser was measured using a PM100A power meter (ThorLabs Inc., Newton, New Jersey). The SHG signal and hepatocyte autofluorescence (AF) were generated using excitation at a wavelength of 800 nm. Backward-directed SHG signals were detected in the range of 371 to 421 nm. Hepatocyte AF was detected in the range of 433 to 660 nm. To account for the fluctuations of the laser power and correct for the scattering effects, we have made reference measurements of the SHG signal generated on the glass–air interface and for each image made a background correction.^26^ The average power incident on the samples was ∼6  mW. A C-Apochromat 40×/1.2 water immersion objective was used for image acquisition. Midline laparotomy was performed to expose the liver. The images of unfixed liver tissues were collected within 15 min of the start of surgical procedures. Ten images were collected for each liver from nonoverlapping fields.

### 2.3 Fluorescence Lifetime Data Analysis

FLIM imaging based on the endogenous fluorescent cofactors is an established approach used to analyze cellular metabolism. The nonphosphorylated form of NADH acts as an electron donor in the mitochondrial electron transport chain. This form of the cofactor is generated during glycolysis and the tricarboxylic acid cycle via the reduction of NAD+. The fluorescence lifetime of NADH depends significantly on the state of the cofactor (whether “free” or “protein-bound”).^27^ FAD linked with protein can exist in two conformations: (1) “closed” or stacked, in which the coplanar isoalloxazine and adenine rings interact through π–π interactions, resulting in very efficient fluorescence quenching, and (2) “open” or unstacked, in which the two aromatic ring are separated from each other.^28^ FAD-containing proteins participate in a variety of metabolic pathways, including electron transport, DNA repair, nucleotide biosynthesis, the beta-oxidation of fatty acids, and amino acid catabolism, as well as the synthesis of other cofactors such as coenzyme A, coenzyme Q, and heme groups.^7^^,^^29^

The FLIM data were analyzed using both bi- and tri-exponential fitting models from a $212\times 212\;{\mu m}^{2}$ imaging area. The fluorescence lifetimes and the relative contributions (free/protein-bound forms of NADH, and bound NADPH) and open/closed conformations of FAD) were calculated for the region of interest by finding the global minimum of $\chi^{2}$ value. The mean values of $\chi^{2}$ and the fluorescence lifetimes in healthy and diseased liver were assessed in the hepatocyte cytoplasm (in which fluorescence arises primarily from mitochondria) by selecting ∼40×40  pixel zones as regions of interest. Nuclei were excluded from these measurements. The FLIM data were processed in SPCImage software (Becker & Hickl GmbH).

### 2.4 TOF-SIMS

Mass spectrometry measurements were performed on a TOF-SIMS 5 instrument (ION-TOF GmbH, Germany) equipped with 30-keV ${\mathrm{Bi}}^{3+}$ primary ions. All the spectra were reordered in spectroscopy mode from a $300\times 300\;{\mu m}^{2}$ area. The primary ion dose density was below the static SIMS limit ($∼3.5\times 1011\text{ ions}/{\mathrm{cm}}^{2}$). At least two different sections of the same pathology and control samples were analyzed. Eighteen mass spectra were recorded for each section. A low-energy electron flood gun was activated to avoid charging of the sample. Secondary ion yields were calculated as the intensity of the corresponding peak of interest normalized to the total ion count using SurfaceLab software (ION-TOF GmbH, Germany). The average ion yields of lipids and amino acids were calculated as mean values acquired from randomly selected areas of different tissue sections both for the pathology and control samples. The saturated fatty acid ion yield was calculated as the sum of the C14:0, C16:0, and C18:0 yields. The polyunsaturated fatty acid yield was calculated as the sum of the C16:2, C18:3, C18:2, C20:3, and C20:4 yields. The unsaturated fatty acid yield was calculated as the polyunsaturated acid yield plus the C16:1 and C18:1 yields.

### 2.5 Morphological Analysis

Liver specimens were fixed in 10% buffered formalin and embedded in paraffin. The 8-μm sections were cut using a microtome (Leica SM 2000; Germany) and mounted on glass slides. Ten cross-sections from each liver were stained with Van Gieson picrofuchsin (VG) to analyze the general histoarchitecture of the tissues, the collagen structure, and histopathological changes. All sections were examined using light microscopy, on a Leica DM1000 system.

### 2.6 Statistical Analysis

A statistical analysis was performed with the help of STATISTICA 64 software, version 10 (StatSoft Inc., Tulsa, Oklahoma). Mean and standard deviation values were used to express the data, whereas the student’s t-test and the one-way ANOVA Bonferroni *post-hoc* test were used to compare the data. Results were considered statistically significant with a p value ≤0.05.

## 3 Results

### 3.1 TPEF and SHG Microscopy of Normal and Diseased Livers

**Fig. 1:**
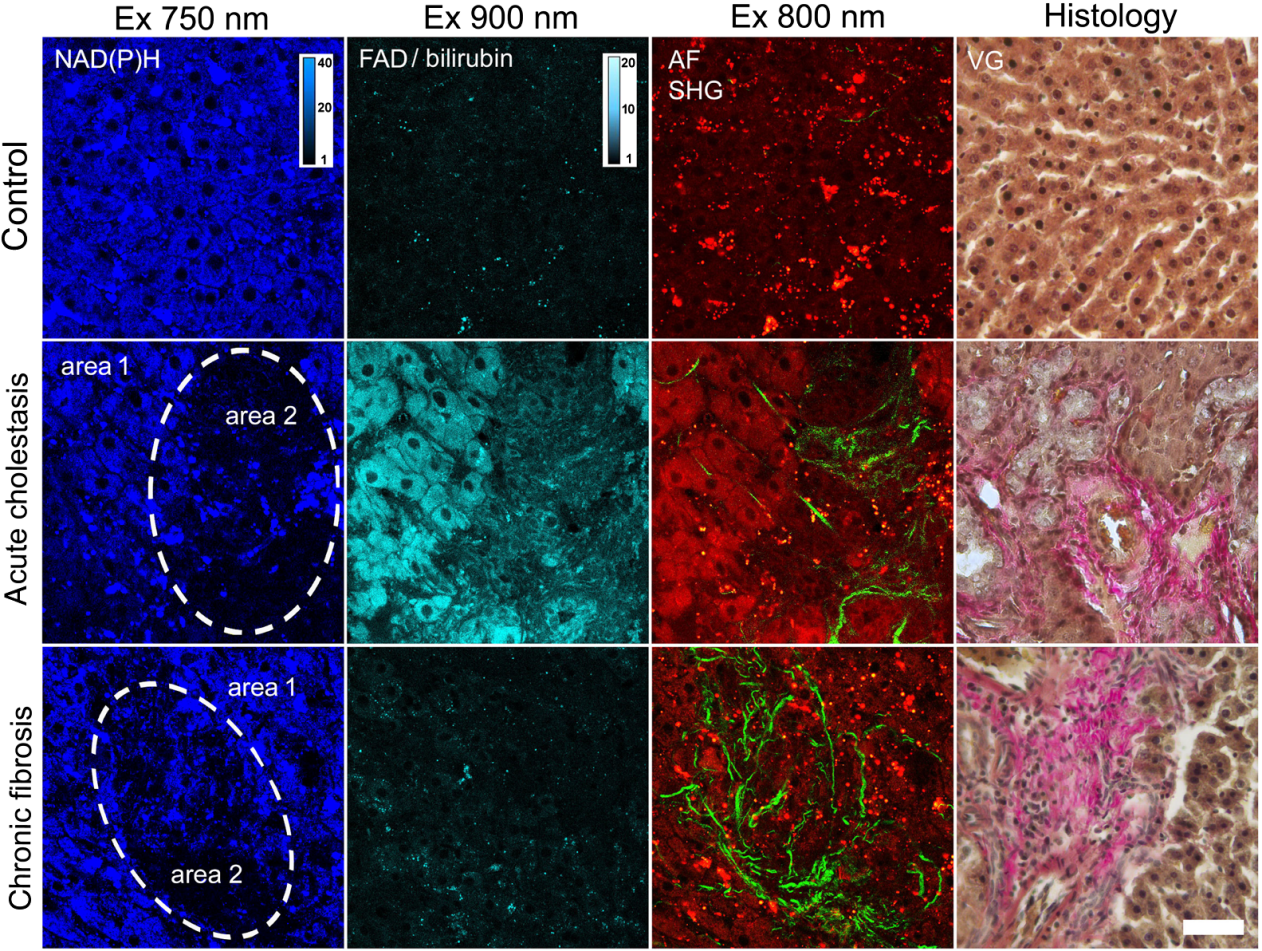
Specific changes in acute cholestasis after 1 week and with chronic fibrosis after 12 weeks. Fluorescence intensity images with morphological data. NAD(P)H: excitation 750 nm, detection 455 to 500 nm; FAD/bilirubin: excitation 900 nm, detection 500 to 550 nm; AF (red): excitation 800 nm, detection 433 to 660 nm; SHG (green): excitation 800 nm, detection 371 to 421 nm. VG: Van Gieson picrofuchsin. Bar: 50  μm.

The cellular structure of the liver could be imaged to a depth of 250  μm below the fibrous capsule using multiphoton microscopy. Wang et al.^30^ had previously found that an imaging depth of 50  μm provided the clearest observations of cellular and subcellular morphology. In normal rat liver, hepatocyte cords showing bright fluorescence of NAD(P)H (primarily from mitochondria) could be distinguished from the dark, blood-filled sinusoids in the fluorescence image. TPEF of FAD was negligible in comparison with NAD(P)H. The signal level in the control was 3±0.3  a.u and 24±0.8  a.u for FAD and NAD(P)H, respectively. The stellate cells were found to be smaller in size and had a strong vitamin A-associated fluorescence. Stellate cells are known to store 80% of vitamin A in the whole body, and vitamin A absorbs light with wavelength of 765±65  nm and could emit strong fluorescence at below 500 nm.^31^ In diseased liver, the hepatocytes lost their normal shape and clear borders. Injury to the hepatocytes could also be observed as reduced fluorescence in the fluorescence images for both cholestasis and fibrosis. It was previously shown that different pathologies (toxic fibrosis, hepatocellular carcinoma, and ischemia) may lead to significantly reduced fluorescence of hepatocytes.^30^ Thus, we have identified two areas for analysis in images of diseased liver: hepatocytes with a high and low NAD(P)H signal (areas 1 and 2, respectively) (Fig. 1).

In the case of acute cholestasis, hepatocyte damage was associated with the accumulation of bile and its pigments, in particular, bilirubin [Table 1(a)]. The highest signal upon excitation at 900 nm, associated with the strong AF of bilirubin and a weak FAD fluorescence, was detected in rat liver on seventh day after the induction of acute cholestasis. The AF signal of NAD(P)H was reduced compared with that of the control liver. At the 3-week time point, the NAD(P)H signal for both areas 1 and 2 had reached a minimum critical value while the FAD/bilirubin signals had returned to their normal values. The peak accumulation of bile in the liver with acute mechanical cholestasis occurred 7 days after the induction of pathology. After 3 weeks, bilirubin could not be detected in the liver. However, toxic damage to the hepatocytes continued to increase to week 3 and had resulted in a significant reduction in the NAD(P)H signal for areas 1 and 2. These typical changes for cholestasis were confirmed by morphological analysis: bile duct expansion and cholangiocyte proliferation. For details about the morphological changes, refer to Supplemental Figs. S1(A)–S1(C). The experimental animals had jaundiced skin and typical urinary symptoms of jaundice.

**Table 1:** TPEF intensity for normal and diseased livers.

(a) Acute cholestasis					
Intensity (a.u.)	Excitation 750 nm [NAD(P)H]		Intensity (a.u.)	Excitation 900 nm (FAD/bilirubin)	
	Area 1	Area 2		Area 1	Area 2
Control	24±0.8	Control	3±0.3		
1 week	19±0.7*	15±1.1***	1 week	14±1.3*	15±1.2*
3 weeks	8±0.3*	2±1.2***	3 weeks	4±0.3*	3±1.8
(b) Chronic fibrosis					
Intensity (a.u.)	Excitation 750 nm [NAD(P)H]		Intensity (a.u.)	Excitation 900 nm (FAD)	
	Area 1	Area 2		Area 1	Area 2
Control	24±0.8	Control	3±0.3		
4 weeks	27±0.7*	12±0.9***	4 weeks	3±0.2	2±0.1**
8 weeks	29±0.8*	16±0.8***	8 weeks	3±0.2	4±0.3***
12 weeks	37±1.1*	12±0.9***	12 weeks	5±0.3*	3±0.1**

* Statistically significant difference from control, p<0.05.

** Statistically significant difference from area 1, p<0.05.

**Fig. 2:**
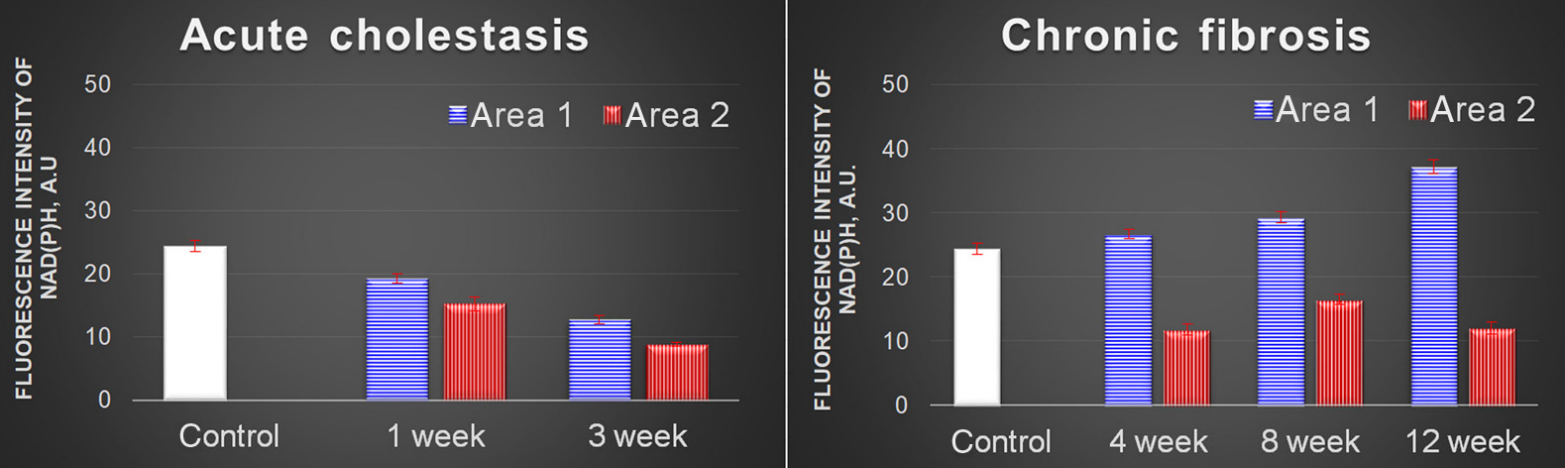
TPEF intensity of NAD(P)H for normal and diseased livers.

For chronic pathology, fibrosis, we observed a different tendency. Normal rat liver of the control group had no SHG signal, indicating an absence of fibrous tissues. Strong and widely spreading SHG signals indicated fibrillar collagen deposition in the rat liver with induced fibrosis. Collagen deposition was observed mainly in the centrilobular region. Hepatocytes with a high NAD(P)H signal but with no collagen fibers around them were identified as area 1. Hepatocytes with a low NAD(P)H signal were detected in the areas of formation of fibrous structures and were marked as area 2 [Table 1(b)]. In contrast with acute cholestasis, the general level of TPEF intensity in the NAD(P)H channel for area 1 increased significantly during the development of the pathology. In area 2, the NAD(P)H signal halved after 1 month and remained at that level for up to 3 months (Fig. 2). The FAD signal intensity did not change dramatically at any time point compared with the control. The changes detected by multiphoton microscopy correlated well with the morphological data.

### 3.2 FLIM Microscopy of Normal and Diseased Livers

**Fig. 3:**
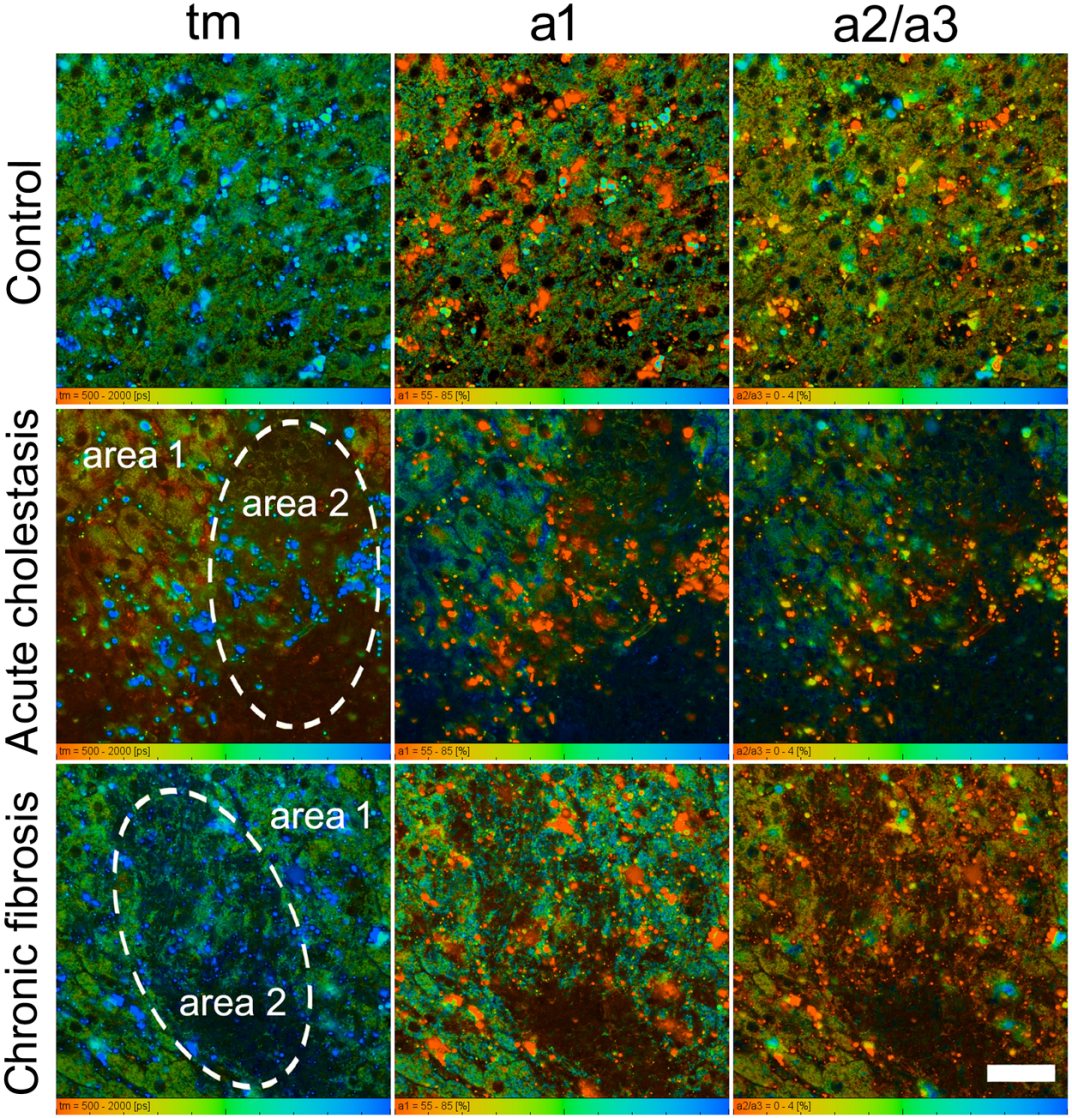
FLIM images of NAD(P)H in liver tissue in acute cholestasis after 1 week and chronic fibrosis after 12 weeks. Amplitude weighted mean lifetime (tm) and the relative contributions of protein-free and protein-bound NAD(P)H for the tri-exponential fitting model. Bar 50  μm.

NAD(P)H fluorescence contains the contributions from free NADH, bound NADH and NADPH which can be involved in the oxidative stress often associated with cellular damage in liver diseases.^32^ To estimate the contribution of NADPH, we have performed an analysis of FLIM data using three-exponential fit of the fluorescence decay curves. Such an approach has been earlier implemented in the work of Meleshina et al.^7^ (Fig. 3). In our case, it was justified by the fact that bi-exponential fit resulted in the increased values of fluorescence lifetimes not only for free NADH (around 530 ps) but also for the significantly increased values of bound NAD(P)H (around 3100 ps) compared with the known ones. Thus it was natural to assume that phosphorylated form makes a contribution to the fluorescence signal. It has been reported earlier that the intracellular fluorescence lifetimes of protein-bound NADH and NADPH are 1500±200 and 4400±200  ps, respectively.^33^ Since the fluorescence lifetime of NAD(P)H when bound to an enzyme is determined by its local environment in the binding site, the NADH and NADPH-binding sites are two of the most highly conserved in all biology.^34^^,^^35^ We have fixed these lifetimes and determined the relative contributions of nonphosphorylated (a2) and phosphorylated (a3) forms of NADH. The FLIM images of FAD were analyzed with the bi-exponential fitting model.

In normal rat liver, the amplitude weighted mean lifetime (tm) of NAD(P)H for the tri-exponential fitting model was determined to be 954 ps. The parameter tm of FAD for the bi-exponential fitting model was 267 ps. The relative contributions of protein-free NADH (a1), protein-bound NADH (a2), and protein-bound NADPH (a3) were 69%, 22%, and 10%, respectively. The parameters a1 (closed form) and a2 (open form) for FAD were 80% and 20%, respectively. Because of the low contribution of bound NADPH, we present the images of a2/a3 ratio to express change between normal and pathological livers.

For acute cholestasis [Table 2(a)], we observed that a1, the contribution of protein-free NADH, had increased in the damage area with a high bilirubin signal after 1 week. The contributions of protein-bound NAD(P)H, a2 and a3, were decreased compared with the control in area 2. The hepatocytes of area 1 showed no changes after 1 week as compared with the control. By week 3, a1,a2, and a3 in area 2 have been restored to normal values close to those of the control. There were still no differences between the contributions of the protein-free and protein-bound NAD(P)H of area 1 after 3 weeks and the control group. In the case of cholestasis, we did not analyze the changes in lifetimes and their contributions for FAD, because the great contribution of bilirubin fluorescence signal excited in the 900 nm (540- to 550-nm emission), complicating the analysis of open/closed FAD contributions.

**Table 2:** Fluorescence lifetimes and the relative contributions of different forms of NAD(P)H and FAD [a1,a2, and a3 for the NAD(P)H tri-exponential fitting model, and a1,a2 for the FAD bi-exponential fitting model] in normal and diseased livers.


(a) Acute cholestasis										
NAD(P)H, tri-exponential fitting model										
t2 and t3 were fixed at 1500 and 4400 ps										
	tm (ps)		t1 (ps)		a1 (%) (protein-free NADH)		a2 (%) (protein-bound NADH)		a3 (%) (protein-bound NADPH)	
	Area 1	Area 2	Area 1	Area 2	Area 1	Area 2	Area 1	Area 2	Area 1	Area 2
Control	954±62		291±10		69±3		22±2		10±1	
1 week	988±176	635±131***	373±25*	241±16***	71±3	81±2***	21±2	14±3***	9±1	6±1***
3 weeks	1051±89	698±155***	376±22*	230±14***	66±1	74±2***	22±2	17±1***	12±1	10±1**
(b) Chronic fibrosis										
NAD(P)H, tri-exponential fitting model										
t2 and t3 were fixed at 1500 and 4400 ps										
	tm (ps)		t1 (ps)		a1 (%) (protein-free NADH)		a2 (%) (protein-bound NADH)		a3 (%) (protein-bound NADPH)	
	Area 1	Area 2	Area 1	Area 2	Area 1	Area 2	Area 1	Area 2	Area 1	Area 2
Control	954±62		291±10		69±3		22±2		10±1	
4 weeks	1168±51*	1333±44***	429±67*	419±109	61±5*	64±4*	25±4	22±3	14±3*	18±2***
8 weeks	1156±52*	1202±50*	285±114	205±93	61±4*	59±5*	26±7	28±5*	13±2*	14±2*
12 weeks	1060±200	1246±220***	431±133	512±132	65±5	61±8*	24±6	28±7*	12±4	13±4
(c) Chronic fibrosis										
FAD, two-exponential fitting model										
	tm (ps)		t1 (ps)		t2(ps)		a1 (%) (closed FAD)		a2 (%) (open FAD)	
	Area 1	Area 2	Area 1	Area 2	Area 1	Area 2	Area 1	Area 2	Area 1	Area 2
Control	267±33		323±52		3048±327		80±2		20±1	
4 weeks	476±80*	589±80*	263±53	302±47	2598±260	2882±382	91±2*	88±2*	9±2*	12±1*
8 weeks	430±64*	494±72*	239±38	263±43	2088±188*	2212±148*	90±2*	88±2*	10±2*	12±2*
12 weeks	540±94*	576±145*	537±191*	436±23*	2850±740	2080±246*	81±6	77±5	19±5	24±5

* Statistically significant difference from area 1, p<0.05.

** Statistically significant difference from control, p<0.05.

Changes in the fluorescence lifetimes and their contributions associated with chronic pathology had other features [Table 2(b)]. After 4 weeks, the a1 of protein-free NADH for both areas 1 and 2 had become lower than in the control. These changes persisted for 12 weeks. The contribution (a1) of closed FAD has significantly increased by week 4 but slowly returned to its normal value by 12 weeks [Table 2(c)]. Changes in a2 (the contribution of open FAD) showed the inverse relationship. During the development of fibrosis, we did not observe any significant differences in the FAD contributions between areas 1 and 2 for all time points.

### 3.3 TOF-SIMS Analysis of Normal and Diseased Livers

**Fig. 4:**
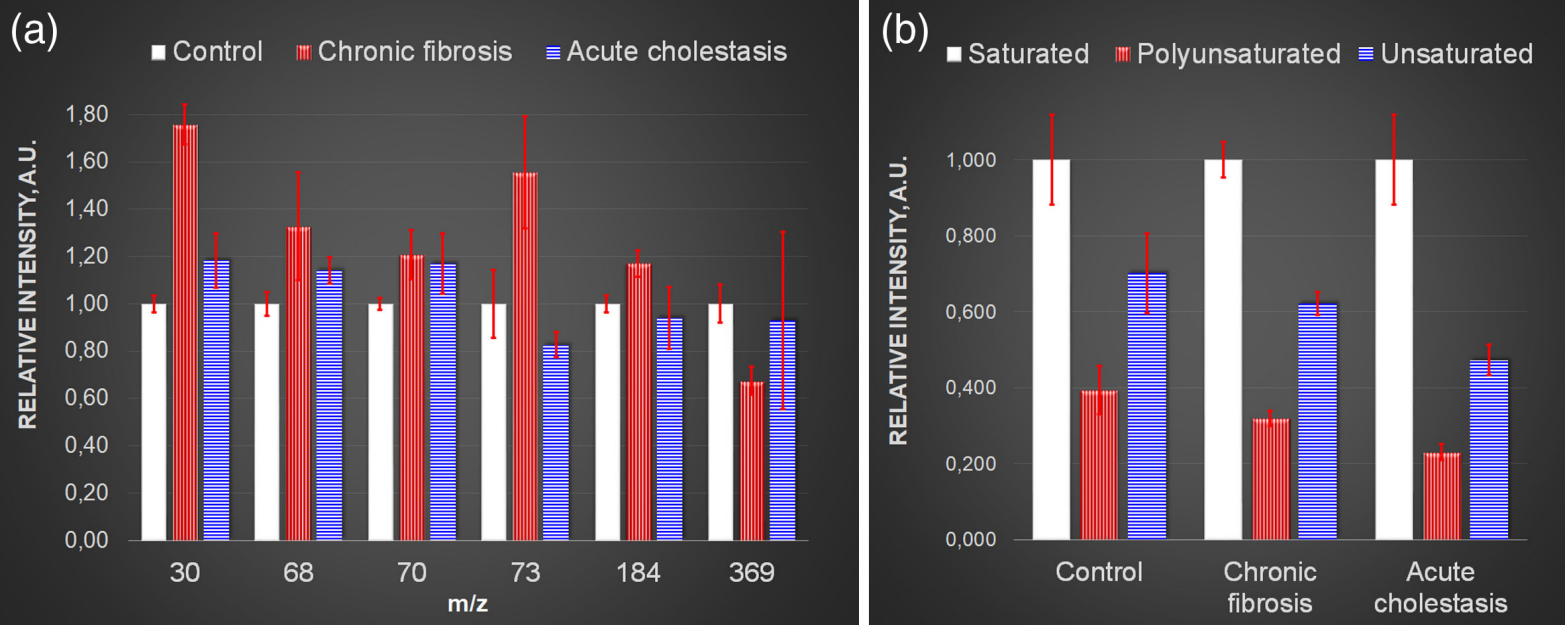
TOF-SIMS analysis of liver tissues for acute cholestasis after 3 weeks and for chronic fibrosis after 12 weeks. Ion yields were normalized to the control sample. (a) Glycine (m/z 30), proline (m/z 68 and 70), arginine (m/z 73), phosphatidylcholine (m/z 184), and cholesterol (m/z 369). (b) Ratio of unsaturated/saturated fatty acids.

TOF-SIMS was used to reveal chemical changes induced by acute cholestasis (after 3 weeks) and chronic fibrosis (after 12 weeks) compared with the control sample. Mainly TOF-SIMS studies are focused on lipid analysis since the technique is sensitive to relatively small molecules (up to 1 to 2000 Da). Nevertheless some information about protein compound can be obtained, based on analysis of the ions attributed to amino acids.^36^^,^^37^ Figure 4(a) presents a comparison of both the lipid and amino acid signals. Ion yields were normalized to the control sample signal for better presentation. The m/z 30 signal, which is mainly attributed to glycine, was significantly enhanced in the fibrosis sample. The proline signal (m/z 68 and 70) is also slightly increased for both the cholestasis and fibrosis sections. The arginine yield (m/z 73) is raised in fibrosis but decreased in the cholestasis sample. It should be noted that glycine, proline, and arginine are known to form collagen and that can explain their increased level for the fibrosis sample.^38^ The phosphatidylcholine and cholesterol levels differed for the control and fibrosis samples. The cholesterol signal level was decreased by 30% compared with the control liver. Figure 4(b) shows the fatty acid compound changes caused by the pathologies. The fatty acid signal is mainly derived from acylglycerols and phospholipids. It is clearly seen that the unsaturated and polyunsaturated fatty acid signals decrease both for fibrosis and cholestasis. While the intensity of decline for the fibrosis samples is within experimental error, the cholestasis samples show a noticeable difference. Hence fatty acid chain saturation occurs during cholestasis progression.

## 4 Discussion and Conclusions

Surgical resection of the liver is currently the only radical treatment for liver tumors. About 13% to 30% of patients can undergo surgical treatment, and the 5-year survival reaches 15% to 60% after liver resection. ^39^ One of the main limiting factors for effective liver resection is the residual critical volume of the liver parenchyma, which is necessary for liver function.^40^^,^^41^ A deficiency of liver parenchyma significantly increases the risk of the small-for-size liver syndrome and of postoperative mortality. The pathogenesis of the small-for-size liver syndrome is not completely understood but is determined by many factors. Important aspects are the initial state of the hepatocytes and their associated pathologies. Clinical methods to assess the initial state of the liver parenchyma (ultrasound, PET, MRI, CT, biochemical blood tests, and clearance tests using indocyanine green and radioactive isotopes) are ineffective and provide only relative guidelines when planning surgery. Multiphoton microscopy with FLIM and SHG, as well as chemical mapping with TOF-SIMS, can be useful tools in determining the structure and function of the liver in both acute and chronic pathologies. Multiphoton microscopy gives information about the intensity and fluorescence lifetimes of the different forms of the intracellular cofactors NAD(P)H and FAD that are involved in the metabolic processes and in oxidative stress associated with cellular damage in liver diseases. NAD(P)H and FAD can be considered as biomarkers for the metabolic activities of cells.^42^^,^^43^ Moreover, we may also evaluate fibrous structures based on the second optical harmonic generation from collagen. Chemical mapping with TOF-SIMS allows a deep analysis of the lipid composition of liver tissue based on the secondary ion spectrum of the samples.^44^ A combination of these techniques provides more complete information about the liver structure and function than could be gained using each method individually.

In earlier works, analysis of liver tissue had already been carried out using multiphoton microscopy. Thorling et al.^21^ characterized liver morphology toward gaining an understanding of changes in the subcellular function in steatotic livers exposed to ischemia-reperfusion injury through a quantitative description of fluorescein distribution obtained by *in vivo* multiphoton microscopy using a physiological pharmacokinetic model. Wang et al. performed systematic multiphoton imaging of normal liver and livers with a range of pathologies (steatosis, fibrosis, hepatocellular carcinoma, and ischemia-reperfusion injury) to assess the potential of multiphoton microscopy for real-time histology and the diagnosis of liver diseases. The authors showed the value of the lifetime measurement and the contributions of the free and bound forms of NADH for the bi-exponential decay model function.^31^ Another work demonstrated the study of cellular oxidative stress in liver injury using multiphoton microscopy.^9^ Fluorescence intensity imaging, FLIM, and sensing probes for glutathione and reactive oxygen species were combined to detect oxidative stress in the livers of living mice. This approach was proposed as a tool for monitoring cellular oxidative stress, to predict drug responses in the most common types of liver injury.^9^

Here, we have presented studies of the liver metabolism, lipid composition, and fibrous structures during the progression of acute and chronic pathologies using fluorescence intensity imaging, FLIM, and TOF-SIMS without any probes or labeling. We hope that our data will help expand understanding of the processes that occur during the development of liver pathology, as well as complement recent works on the study of the structure and function of the liver using multiphoton microscopy.

In acute and chronic pathology, we can distinguish two areas in the damaged liver. The area with the most obvious damage (area 2) caused by the accumulation of bile or the formation of fibrous structures is characterized by a reduced NAD(P)H signal. In the case of cholestasis in combination with a reduction in total fluorescence intensity of the NAD(P)H, we can observe an increase in the contribution of protein-free NADH and a decrease in the contributions of the protein-bound forms of NAD(P)H. Changes in these parameters allow us to conclude that there has been a general decrease in the metabolic activity of the hepatocytes. Moreover, a change in the ratio of the protein-free and protein-bound forms of NADH may indicate a relative increase in the extent of anaerobic processes (glycolysis) and a decrease in the intensity of oxidative phosphorylation (OXPHOS). An increase in the relative contribution of protein-free NADH (*a*1) is associated with a lower level of OXPHOS and a higher level of glycolysis. NADPH is involved in biosynthetic processes, in particular, of fatty acids and cholesterol and the functioning of the glutathione reductase system. Thus, the reduction of protein-bound NADPH (a3) may indicate that the level of such synthetic processes had fallen.^7^^,^^45^ These results were confirmed by TOF-SIMS analysis that indicated a noticeable reduction in the signal from unsaturated and polyunsaturated fatty acids in the case of acute cholestasis. In the surrounding tissue (area 1), we can also see a decrease in the total fluorescence intensity of NAD(P)H but not so dramatically as in area 2. The contributions of protein-free and bound NADH showed no differences from the control group. Thus, in the border area, we may be observing a decrease in the total metabolic activity but without a change in the metabolic pathways occurring. A peak of bile accumulation in the liver tissue was detected 1 week after the induction of cholestasis, as shown by the bright fluorescence signal of bilirubin upon excitation at 900 nm. After 3 weeks, the amount of bilirubin had returned to a value close to normal. However, toxic damage caused by the accumulation of bile acids peaked. This was expressed in a decrease in the total fluorescence intensity indicating changes in metabolic activity to critical levels.

During the progression of chronic pathology, we see another tendency in hepatocyte metabolic changes. Toxic liver damage by alcohol is expressed by the formation of fibrous collagen structures. Hepatocytes surrounded by collagen fibers were also characterized by a reduced signal from NAD(P)H (area 2). But, unlike the acute pathology, the contribution of protein-free NADH (a1) was down during fibrosis progression, whereas a3 was increased. Analysis of the total fluorescence intensity of the NAD(P)H suggests that the metabolic rate in area 2 had decreased. The increase in a3 (the contribution of the phosphorylated form of NADPH) in area 2 may have taken place due to oxidative stress in the damaged hepatocytes. It is known that NADPH is needed for the biosynthesis of fatty acids and cholesterol in the cytosol. But NADPH is also a required cofactor for the glutathione and thioredoxin systems used to neutralize the reactive oxygen species that result from oxidative stress in the cytosol and in the mitochondria.^46^ Thus, oxidative stress evokes a metabolic adaptation that favors increased NADPH and decreased NADH.^46^^–^^48^ Both the total NAD(P)H fluorescence intensity and the a3 of the border tissue (area 1) were raised during the 3 months of fibrosis progression. Growth of the NAD(P)H signal and the contribution of the phosphorylated form, NADPH, in area 1 may indicate an increase in metabolic processes due to the switching-on of protective mechanisms and the biosynthesis of fatty acids in response to the development of chronic pathology. These results were also confirmed by TOF-SIMS. The TOF-SIMS analysis showed an increase in the phosphatidylcholine signal in fibrotic liver. The fatty acid signal is known mainly to result from acylglycerols and phospholipids, in particular, phosphatidylcholine. Expression of fatty acid biosynthesis can be observed throughout the body, with the most prominent expression in the liver, brain, and abdominal adipose tissue, where energy storage is important for cell survival during periods of physiological or pathological stress.^49^^,^^50^ In chronic fibrosis, we also observed a lower total cholesterol level (decreasing by 30% compared with the control liver) as shown by the TOF-SIMS analysis. Much research has shown that chronic liver diseases strongly impair lipid metabolism, with hypocholesterolemia as a common finding in cirrhosis.^51^

Thus, we have analyzed the progression of acute and chronic liver pathology using multiphoton microscopy with FLIM and SHG modes together with TOF-SIMS composition analysis to obtain new data about pathological changes in hepatocytes at the cellular and molecular level. All of these techniques enable us to study cellular metabolism, lipid composition, and collagen structure without sample staining or the incorporation of fluorescent or other markers, meaning that these methods are potentially applicable to clinical use. Furthermore, the combination of multiphoton microscopy and mass spectrometry provides a more complete information about the liver structure and function than what could be assessed using either method individually. The data can be used both to obtain new criteria for the identification of hepatic pathology and to develop a rapid technique of liver quality analysis in order to plan surgery and to help avoid postoperative liver failure in clinic.
