# Supplementary material for: Metabolic imaging and secondary ion mass spectrometry to define the structure and function of liver with acute and chronic pathology
Source: J Biomed Opt. 2019 Dec 17;25(1):014508. doi: 10.1117/1.JBO.25.1.014508 (PMC7008498; doi:10.1117/1.JBO.25.1.014508)
Supplement: Supplementary file 1 [file JBO_025_014508_SD001.pdf]

Supplementary Material

A (control, 10x)

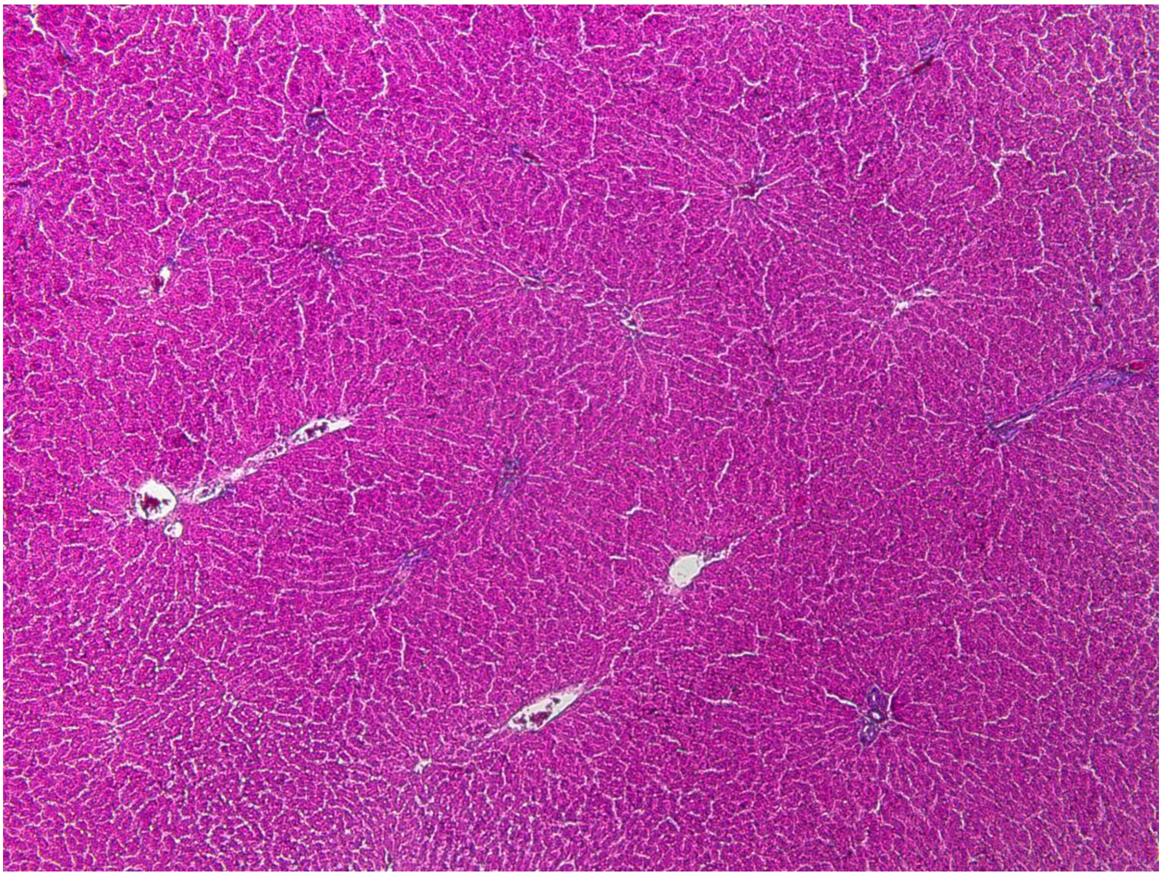

B (acute cholestasis 1 week, 10x)

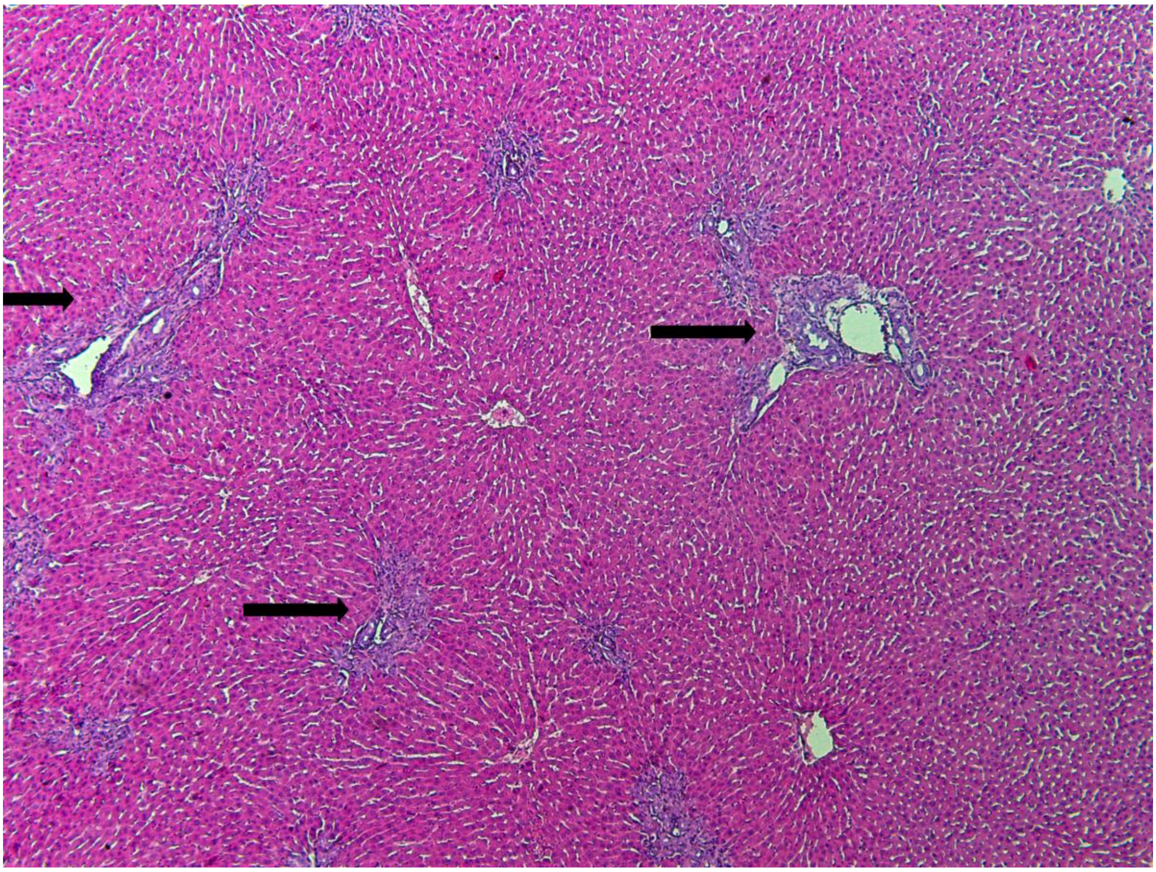

C **(acute cholestasis 3 weeks, 10x)**

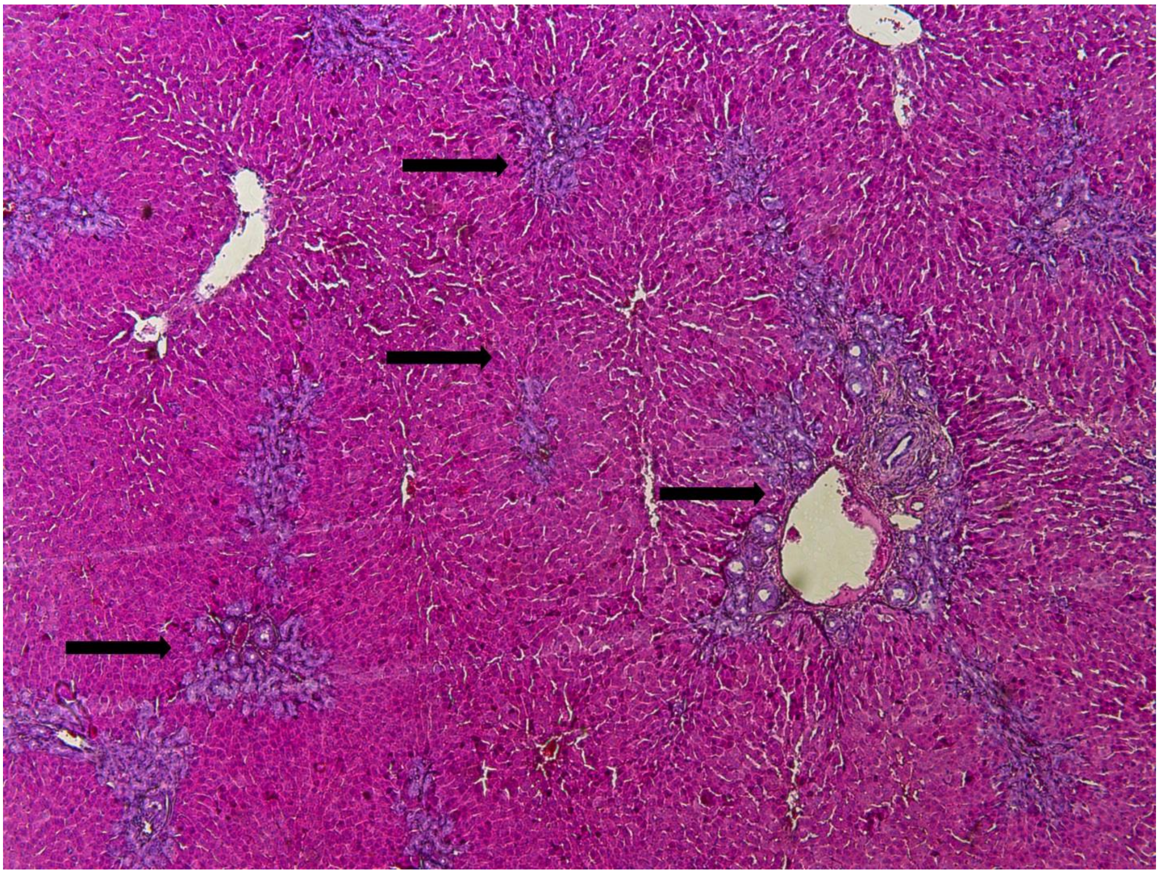

Fig S1. Typical changes for cholestasis. The arrows indicate bile duct expansion and cholangiocyte proliferation. H&E staining.
